# Screw-Dislocation-Driven Growth Mode in Two Dimensional GaSe on GaAs(001) Substrates Grown by Molecular Beam Epitaxy

**DOI:** 10.1038/s41598-019-54406-5

**Published:** 2019-11-28

**Authors:** Nhu Quynh Diep, Cheng-Wei Liu, Ssu-Kuan Wu, Wu-Ching Chou, Sa Hoang Huynh, Edward Yi Chang

**Affiliations:** 1 Department of Electrophysics, College of Sciences, National Chiao Tung University, 1001 University Road, Hsinchu, 30010 Taiwan, R.O.C.; 2 Department of Materials Science and Engineering, College of Engineering, National Chiao Tung University, 1001 University Road, Hsinchu, 30010 Taiwan, R.O.C.; 3 International College of Semiconductor Technology, National Chiao Tung University, 1001 University Road, Hsinchu, 30010 Taiwan, R.O.C.; 4 Present Address: School of Physics and Astronomy, Cardiff University, Cardiff, CF24 3AA United Kingdom

**Keywords:** Two-dimensional materials, Raman spectroscopy

## Abstract

Regardless of the dissimilarity in the crystal symmetry, the two-dimensional GaSe materials grown on GaAs(001) substrates by molecular beam epitaxy reveal a screw-dislocation-driven growth mechanism. The spiral-pyramidal structure of GaSe multi-layers was typically observed with the majority in *ε*-phase. Comprehensive investigations on temperature-dependent photoluminescence, Raman scattering, and X-ray diffraction indicated that the structure has been suffered an amount of strain, resulted from the screw-dislocation-driven growth mechanism as well as the stacking disorders between monolayer at the boundaries of the GaSe nanoflakes. In addition, Raman spectra under various wavelength laser excitations explored that the common *ε*-phase of 2D GaSe grown directly on GaAs can be transformed into the *β*-phase by introducing a Se-pretreatment period at the initial growth process. This work provides an understanding of molecular beam epitaxy growth of 2D materials on three-dimensional substrates and paves the way to realize future electronic and optoelectronic heterogeneous integrated technology as well as second harmonic generation applications.

## Introduction

Two-dimensional (2D) group IIIA-monochalcogenide family has become a rising star in the field of condensed matter physics in recent years, offering opportunities for engineering novel optical and electrical device applications. In particular, gallium selenide (GaSe), a typical 2D semiconductor, is widely recognized as a potential candidate for electronics, nonlinear optics, terahertz generation, especially photo-detection applications with low dark current, high photoresponsivity, and ultra-fast response time^1–5^. The layers of bulk GaSe crystals are weakly coupled to each other by van der Waals force while atoms in each layer connected via covalent bonds. As a member of 2D material family, the feature of crystal structure of bulk GaSe is classified into four different polytypes (phases) as *ε*-(2R), *β*-(2H), *γ*-(3R), and *δ*-(4H) based on staking sequence between adjacent layers, resulting in a variety of electronic and optical properties^6,7^. Experimentally, *ε*-GaSe and *β*-GaSe are the most observable phases; both consist of two layers in their unit cell. However, the symmetric group of *ε*-GaSe phase belongs to *D*^1^_*3h*_ while that of the latter one is *D*^4^_*6h*_, corresponding to distinctions in optical behaviors between them^8^. Indeed, it is well-known that bulk *ε*-GaSe possess two-order nonlinear-optical property. Bilayer *ε*-GaSe phase exhibits a strong second harmonic generation (SHG) signal while there is no SHG behavior observed on *β*-GaSe phase^9^. Therefore, the synthesis and the phase controllability of large-scale 2D GaSe have attracted more and more attention. Several fabrication approaches have been proposed such as exfoliation, chemical and physical vapor deposition (CVD and PVD), molecular beam epitaxy (MBE) and so on^10–12^. Among them, MBE is an ideal method to achieve high single-crystalline quality and free-contamination 2D GaSe materials on 3D-crystal substrates. Recently, a variety of 3D-substrates that has the same six-fold surface symmetry with GaSe such as GaAs(111)B^13,14^, Si(111)^15,16^, c-sapphire^17^, and GaN^8^ was chosen to realize single-crystalline 2D GaSe using MBE. However, phase formation and growth mechanism of GaSe have been not considered comprehensively in most of these reports. Moreover, lack of studies on quasi-van der Waals epitaxy (vdWE) of GaSe grown on commercial substrates that have inequality of surface symmetry to GaSe such as Si(001) and GaAs(001) results in inflexibility in controlling crystal quality, area, especially, phase of the materials.

In this study, we report the epitaxial growth of the 2D-3D hybrid structure of 2D GaSe grown on 3D GaAs(001) substrates by MBE. Large-scale spiral-pyramidal structure of GaSe multilayers was observed from atomic force microscopy (AFM) images as a result of screw-dislocation-driven (SDD) growth mode. The comprehensive analyses including high-resolution X-ray diffraction (HR-XRD), Raman scattering, temperature-dependent photoluminescence (PL), and high-resolution transmission electron microscopy (HR-TEM) exposed that the *ε*-phase dominance and the strains of the GaSe multi-layers were originated from the SDD growth mode. Moreover, we found that the dominant ε-phase of GaSe thin films can be tuned to *β*-phase as a specific Se-pretreatment flux applied at the GaSe/GaAs interface. The results presented in this study are imperative in term of providing information about epitaxial MBE growth technique of 2D/3D hybrid structures and exploring phase controllability of the GaSe material.

## Results and Discussion

Spotty RHEED patterns of the substrate surface taken with the incident electron beam parallel to the [110] and [100] substrate directions after the completion of thermal oxide desorption process were observed as shown in Fig. S2(a,b), respectively. The spotty pattern of the rough GaAs surface results from the very top layer desorption, where the existence of Ga-droplets on the surface was ruled out by our AFM study^18,19^. For non-Se-pretreated process, within a minute from starting GaSe deposition, the spotty pattern in Fig. S2(a) was turning quickly into blurry long streaks and became sharp-streaky pattern without any polycrystalline rings when the growth time increased as can be seen in Fig. S2(c). This streaky pattern repeated in every 60° in-plane substrate rotating and was preserved until the growth finishing, indicating the six-fold symmetry of the high-crystalline GaSe layer. Fig. S2(e) notices that the streak intervals in Fig. S2(d) taken after 30° azimuthally rotating from Fig. S2(c)’s position are $\sqrt{3}$ times shorter than that in Fig. S2(c). In other words, the RHEED patterns shown in Fig. S2(c,d) are attributed to those taken from the $[1\bar{1}00]$ and $[1\bar{2}10]$ azimuths of the single-crystalline GaSe layer, respectively^13,18,20^. These RHEED patterns remained unchanged until the growth finishing. The analyzed RHEED results demonstrated that the single-crystalline GaSe(0001) were directly deposited on the (001) oriented GaAs substrates with its $[1\bar{1}00]$ axis parallel to the [110] axis of GaAs substrate as illustrated in Fig. S2(f).

**Figure 1:**
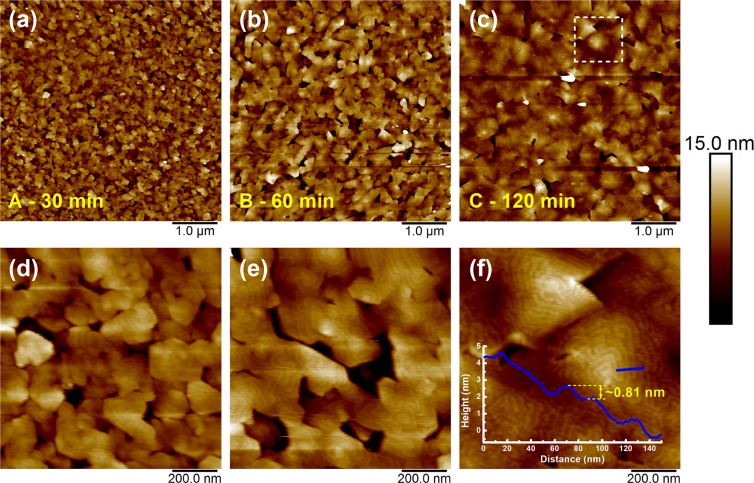
Surface morphologies of GaSe samples A, B, C grown on GaAs(001) substrates by MBE.

Surface morphological evolution of the GaSe samples in case of non-Se-pretreated approach with various growth times is shown in Fig. 1. For 30 min. growth, the GaSe layer was consisted of a large number of small-size GaSe flakes with random shape and orientation as can be easily observed in Fig. 1(a), where its average lateral size was around 120 nm with a surface coverage of ~95%. As increasing the growth time to 60 min., these flakes developed laterally in size and coalesced each other, resulting in a drastic increase in the size of GaSe flakes with an average size of ~500 nm as shown in Fig. 1(b). Figure 1(d,e), the higher magnified AFM image of these samples, showed clearly the spiral configuration of the GaSe surface. The lateral coalescence of GaSe flakes had strongly progressed as further deposition, leading to form a large number of spiral pyramid-like shapes on the surface with the lateral dimension of their triangular based planes ~750 nm for 120 min. growth as can be seen in Fig. 1(c,f). The specific morphology displayed in Fig. 1(f) is a fascinating feature as observed in other 2D materials deposition, where the growth process is driven by screw-dislocation mechanism^21–24^. At the initial nucleation of the growth, a large number of screw defects acted as dislocation centers was nucleated due to the rough and high-density of dangling bonds GaAs surface during thermal-desorption as well as the dissimilar symmetry between GaAs and GaSe nanoseeds, promoting the formation of spiral nanoflakes at random size and shape as well. As further extending growth time, the effect of the substrate surface was gradually released, these nanoflakes were enlarged and merged together, resulting in the emergence of multiple slip planes, and finally initiating the construction of typical spiral pyramid-like morphology^25–27^. The appearance of truncated pyramids observed in Fig. 1(f) has not been clearly understood. Overall, these pyramids are usually composed of several GaSe monolayer stacked spirally together in 0° alignment, where a monolayer thickness was confirmed to be ~0.81 nm as shown in the inset of Fig. 1(f). This morphological characteristic implies that the GaSe stacking orders is AA stacking configuration, which belongs to the noncentrosymmetric *D*^*1*^_*3h*_ group and is usually observed in other 2D materials driven by SDD growth mode^22,25,27^. The signatures presented here claim that the phase of GaSe layers could be *ε*-phase^6^.

**Figure 2:**
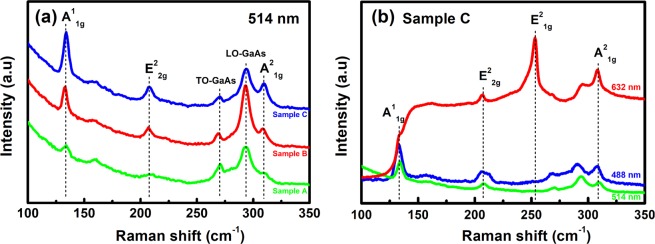
(**a**) Raman spectra of GaSe samples A, B, C under 514 nm laser excitation and (**b**) Raman spectra of sample C taken by various laser excitation wavelengths.

To explore the statement above, Raman spectra of these samples were conducted using various laser excitation wavelengths. Figure 2(a) shows the Raman spectra measured under 514 nm laser excitation of three non-Se-pretreated samples with various growth times (sample A, B, and C). Three Raman-active modes of GaSe were observed in all samples^28,29^. The A^1^_1g_, E^2^_2g_, and A^2^_1g_ modes located at ~133.2 cm^−1^, ~207.0 cm^−1^, and ~307.9 cm^−1^, respectively. As increasing growth time, these peaks had become more intense while their energy position did not shift. For further investigation, Raman spectra of sample C under different excitation wavelengths were carried out, as shown in Fig. 2(b). Interestingly, its Raman spectrum under 633 nm laser excitation appeared an additional strong peak located ~253.0 cm^−1^ with a shoulder at ~246.0 cm^−1^ which was undetectable as excited by both 488 nm and 514 nm laser wavelengths. It is claimed that the 253.0 cm^−1^ line comes from LO-TO splitting of E’ mode which only observed in the *D*_*3h*_-symmetric GaSe structure (ε-phase). Moreover, the previous literature also stated that the appearance of unusual Raman active modes at ~246.0 cm^−1^ and ~253.0 cm^−1^ were due to near-resonant effect in Raman scattering when using the laser excitation which energy approached closely to the electronic transition energy of the sample^30,31^. Thus, it suggests that the emergence of a strong 253.0 cm^−1^ peak with a shoulder around 246.0 cm^−1^ in the Raman spectrum of sample C under 633 nm wavelength excitation are attributed to the near-resonant Raman peaks. The features expressed above should be extensive evidence to prove that the *ε*-phase is dominant in our GaSe epitaxial layers^1,12,30^.

**Figure 3:**
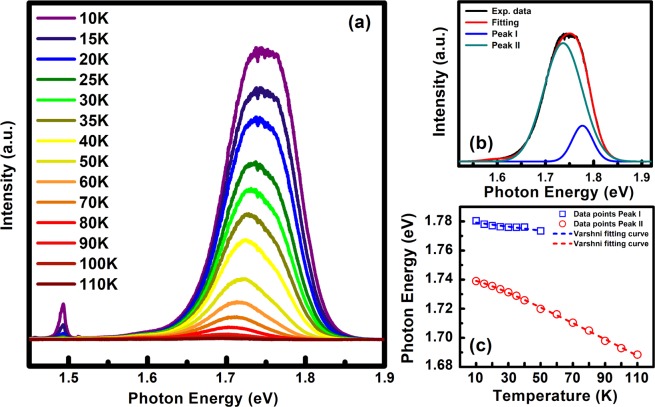
(**a**) PL spectrum as a function of temperature. (**b**) Multi-peak fitting of PL spectrum at 10 K. (**c**) Photoemission energy (blue squares and red circles) as a function of temperature. The dashed lines represent the fitting curve based on the empirical Varshni equation.

**Figure 4:**
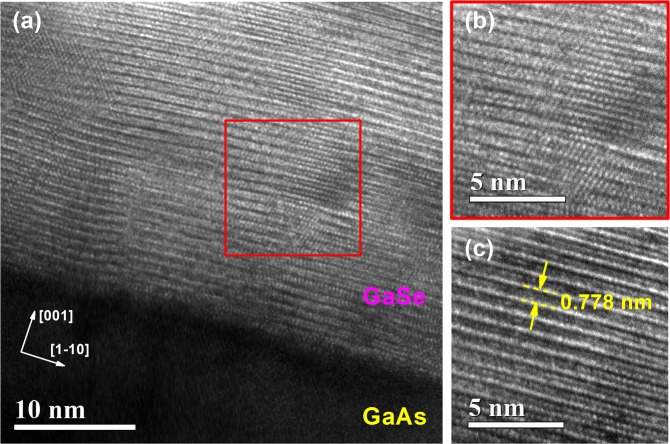
(**a**) HR-TEM image of GaSe thin film grown on GaAs(001). High-magnified TEM image exhibited (**b**) the stacking disorders between layers and (**c**) the layered-structure of GaSe with the average thickness of monolayer is ~0.778 nm.

To study more detail on the optical properties as well as the bandgap energy of GaSe grown on GaAs substrates, temperature-dependent PL spectra of sample C were studied. PL spectra at different temperature of sample C exhibited a strong broad peak centered at around 1.75 eV, as shown in Fig. 3(a). The peak near 1.5 eV is attributed to the near band edge emission of the GaAs substrate^32,33^. The main peak at 1.75 eV is composed of two peaks (denoted as peak I and II) with an energy separation of about 40 meV between them, as illustrated in Fig. 3(b), were attributed to the free exciton and bound exciton^34^. In Fig. 3(a), an expected energy redshift of the optical features (about 0.35 meV/K) and degradation of PL intensity as increasing the temperature was observed, in agreement with the temperature dependence of bandgap of GaSe and other semiconductors reported in the literature^35–37^. The good agreement between experimental data points and the Varshni-fitting curve shown in Fig. 3(c) demonstrated that the bandgap energy of the GaSe thin film grown on GaAs should be around 1.75 eV, ~270 meV smaller than that of the bulk (~2.02 eV) and other exfoliated GaSe thin films reported recently^34,38,39^. This drastic decrease in bandgap was also observed in the intentionally strained (wrinkled) GaSe^37^. Furthermore, nano-scale strain-induced bandgap variation was also investigated in the mono-layer WSe_2_ grown on the intentionally fabricated μm hole by cathode-luminescence^40^. In our study, the spiral-pyramidal morphology of GaSe multi-layers is a result of SDD growth mechanism, recognizing as a major contributor to the strain-induced abnormal bandgap. The SDD growth mode is a climbed-up process starting from the screw dislocation cores, which requires the presence of slipped planes (screw defects) in the initial layer, if not an in-plane growth mode will be introduced^22^. Because of the large strain at the dislocation core, an amount of lattice strain could be presented in our GaSe thin films. Herein, the possibility of strain induced by the lattice mismatch between GaSe layer and the GaAs substrate is ignorable because of the similarity in either 3D-spiral-pyramidal morphology or Raman spectrum or PL emission between GaSe on GaAs and GaSe on GaN/sapphire as further investigated in Fig. S3. Moreover, this lattice-mismatched strain could be relaxed due to the van der Waals epitaxy^41^, as reported in previous papers by Keiji Ueno *et al*. for GaSe on GaAs^13^ or by X. Yuan *et al*. for GaSe on mica^1^. Thus, we argue that the strong redshift in PL peak was the result of SDD growth. Cross-sectional HR-TEM micrographs shown in Fig. 4 exposed that the GaSe thin film is composed of the GaSe multi-flakes, where stacking disorder between layers at the flake boundaries is easily noticed in Fig. 4(b). Moreover, the separation between atoms in an atomic layer was estimated around 0.34 nm in agreement with other previous reports^8,17^, while the average monolayer thickness of 0.778 nm observed from the HR-TEM image in Fig. 4(c) is slightly smaller than the theoretical bulk value^29^. It means that the GaSe film might be suffering an out-of-plane compressive strain. HR-XRD 2θ scans were carried out carefully in order to identify the position of the (004) diffraction peak as can be seen in Fig. S4. The expected shift of (004) GaSe film peak to the higher 2θ-angle was observed, demonstrating an estimation of the excessive out-of-plane compressive strain of ~0.25% in our GaSe thin film. A comparison of the Raman spectrum of GaSe bulk sample and sample C was also performed as showed in Fig. S5. The result exhibited the strong redshift (~6 cm^−1^) of in-plane vibration E^2^_2g_ mode in the GaSe film as referring to the bulk sample, indicating an existence of the overall substantial in-plane tensile strain in our thin film. Consequently, our samples were simultaneously suffering both out-of-plane compressive strain and in-plane tensile strain with different magnitudes as a result of the SDD growth mechanism, leading to the observation of the strong redshift PL emission energy^42,43^. Due to the distinct feature of 2D materials grown by SDD growth mode, there are very few reports on the empirical formula or theoretical model to extract quantitatively the lattice strain of the sample based on either PL or Raman shifting of SDD-strained GaSe; thus, it is hard to obtain a correspondence in term of lattice strain degree between PL, Raman, TEM, and XRD measurements.

**Figure 5:**
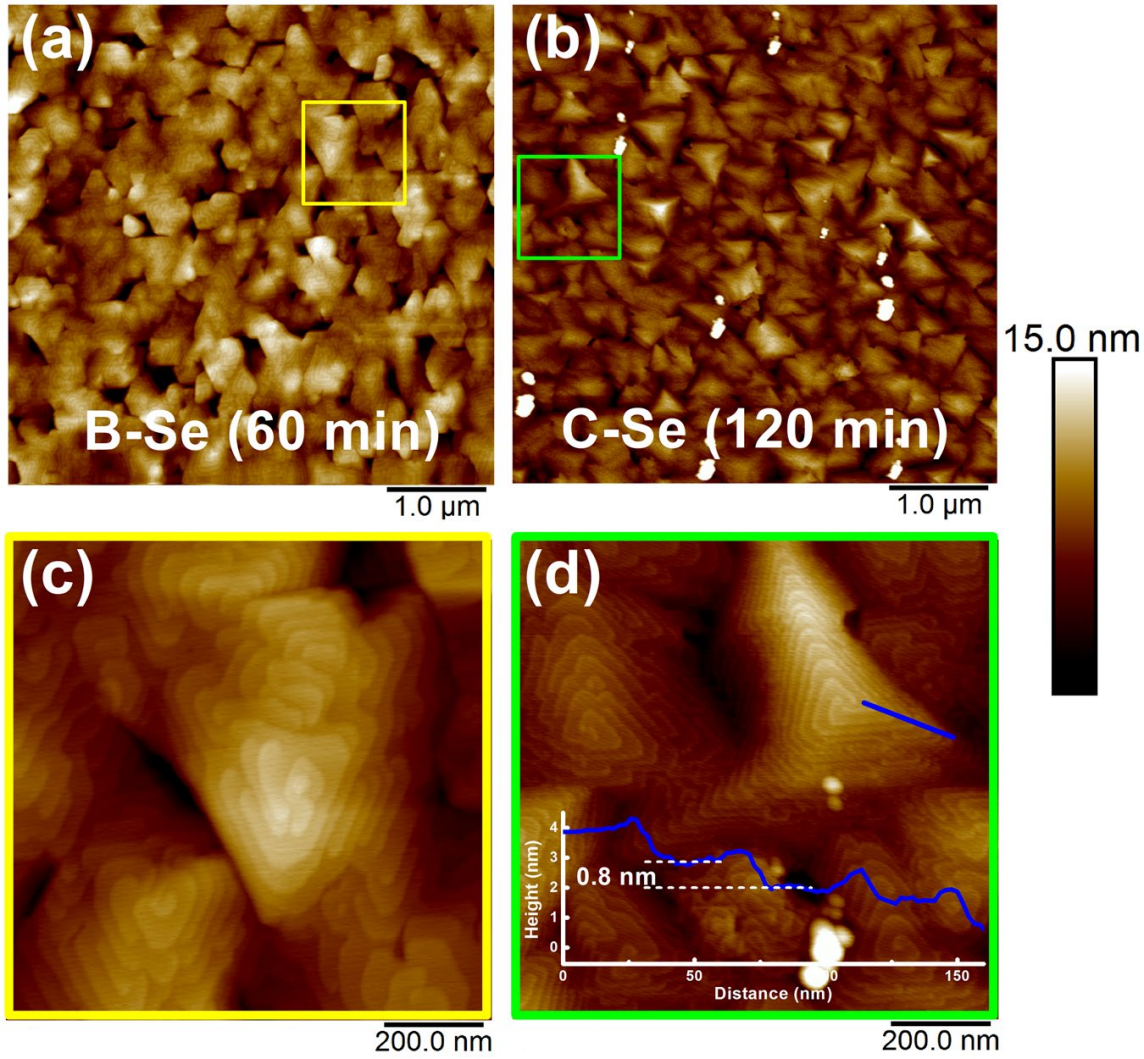
AFM images of Se-pretreated GaSe samples grown at 60 min. and 120 min.

**Figure 6:**
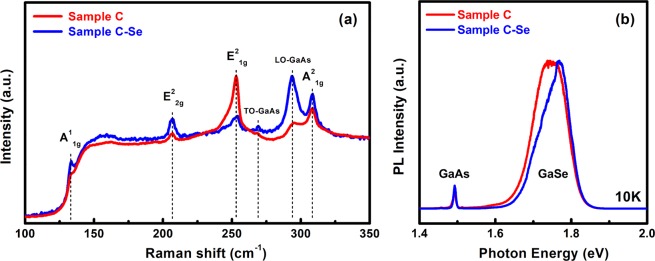
A comparison in (**a**) Raman spectrum taken by 633-nm laser excitation and (**b**) PL emission at 10 K between with and without Se-pretreatment GaSe/GaAs(001).

The effect of Se-pretreatment on the morphology is demonstrated in Fig. 5. The typical spiral-pyramidal morphology of Se-pretreated GaSe thin films has an average step height of ~0.8 nm in sample C-Se, which corresponds to the monolayer stacking configurations. Sample C-Se exhibited a slightly increase in surface roughness as compared to that of sample C (Fig. 1(c)) due to the increase in the GaSe pyramid density, where its lateral size statistically slightly enlarged to 850 nm (Fig. 5(b)). Moreover, the appearance of the blurry extra lines in between spotty lines on the RHEED pattern (Fig. S6(a)) of the Se-pretreated GaAs surface is attributed to the existence of a Se-passivated layer on GaAs surface. Figure 5(d) showed the simultaneous presence of single, double, triple and multi-spiral outlines, this is presumably due to the mixed phase between *ε*-GaSe and *β*-GaSe in the layer^25^. Raman spectrum of sample C-Se was carried out by using 633-nm laser excitation as can be seen in Fig. 6(a). There is no shifting relatively in the position of all Raman active modes between with and without Se-pretreated sample. However, it is interesting that the Raman intensity of the main 253.0 cm^−1^ E^2^_1g_ mode of sample C-Se displayed a significant reduction of ~4 times as compared to that of sample C, while the rest of the common Raman modes (A^1^_1g_, E^2^_2g_, and A^2^_1g_) of sample C-Se was relatively enhanced. As mentioned above, the principal difference between the *β*-GaSe and *ε*-GaSe Raman spectra is the appearance of an additional mode at approximately 253.0 cm^−1^ ^44^. The evolution in Raman intensity of sample C-Se suggests that the growth of GaSe preferred *β*-phase to *ε-*phase. This behavior seems likely an incomplete transition from *ε-* to *β*-phase when the Se-pretreatment process was applied and leads to a mixture of phases in the layer. PL spectrum of sample C-Se measured at 10 K reveals a multiple-line PL emission at the same energy range in comparison to that of sample C (Fig. 6(b)). However, the relative intensity of each emission line between sample C and C-Se is drastically changed. Further PL investigations should be addressed to clarify whether this behavior corresponds to the dominance of *β*-GaSe phase in sample C-Se.

In conclusion, we have demonstrated the MBE epitaxial growth of 2D GaSe on GaAs(001) substrates even though there is an extreme dissimilarity in their crystal symmetry. The evolution of GaSe morphology with growth time and *in-situ* RHEED monitoring provides an understanding of the screw-dislocation-driven growth mechanism of the materials. The studies on Raman spectra figured out the majority of *ε*-phase GaSe in our samples, which was verified by the appearance of the Raman active mode E^2^_1g_ (~253.0 cm^−1^) under only 633 nm laser excitation and has high potential in the field of SHG applications. A strong redshift bandgap energy ~1.75 eV observed in the PL spectra of the GaSe multi-layers are results of strains, originated from screw-dislocation-driven growth mode. The results also reveal that the Se-pretreatment process could be the pathway to transform the phase of GaSe grown on GaAs from *ε*-GaSe into *β*-GaSe phase.

## Methods

The growth of 2D layered GaSe on (001) oriented GaAs substrate was carried out in an SVTA-MBE system operated at a base pressure of 1.0 × 10^−10^ torr with standard Knudsen cells to evaporate high-purity (6N) gallium (Ga) and selenium (Se) elements. The GaAs substrate was cleaned by acetone, rinsed with de-ionized water and dried with nitrogen gas in sequence prior to loading into the MBE chamber through a load-lock component. Firstly, the substrate was heated up to 150 °C and kept at this temperature in 30 minutes for baking. The chamber temperature was then ramped up to 600 °C under an ultra-high vacuum (UHV) of ~7.0 × 10^−9^ torr to ensure surface native oxides removing completely which was monitoring by the *in-situ* reflective high-energy electron diffraction (RHEED). In the next step, the growth was performed into two different approaches including without and with introducing a Se-pretreatment process for 15 min. under a Se partial pressure of 7.76 × 10^−7^ torr as illustrated in Fig. S1. All growth conditions of GaSe thin films were kept the same in both approaches. The Ga and Se source shutters were released simultaneously where their beam equivalent pressure (BEP) during the material growth were 1.39 × 10^−7^ and 7.76 × 10^−7^ torr, respectively, corresponding to a Se/Ga flux ratio of 5.6. The GaSe deposition was terminated by closing both of source shutters and the sample was then cooled down to 100 °C prior to loading out of the chamber. A set of three non-Se-pretreatment samples was deposited with different growth times including 30 min. (denoted as sample A), 60 min. (B), and 120 min. (C). For Se-pretreatment approaches, B-Se and C-Se were assigned to the sample grown with 60 min. and 120 min., respectively. All the growth parameters of GaSe epitaxial layers are addressed in Table 1. *In-situ* RHEED was employed to monitor the sample surface during the whole of the material growth process. Surface morphology and crystal quality of the GaSe samples were carefully examined by AFM Veeco D3100, XRD Bede D1, and HR-TEM Cryo JOEL JEM 2010. Raman spectra using LabRam iHR550 HORIBA spectrometer at three different laser excitation wavelengths (488, 514, and 633 nm) as well as temperature and power-dependent PL using 325-nm continuous wavelength (CW) laser excitation were acquired to investigate comprehensively the structural and optical characteristics of GaSe thin films.

**Table 1:** MBE growth parameters of 2D layered GaSe materials on GaAs (001) substrates.

Sample ID	A	B	C	B-Se	C-Se
Se-pretreatment	—	—	—	15 min. at 600 °C	15 min. at 600 °C
Growth time	30 min.	60 min.	120 min.	60 min.	120 min.
Growth temperature	425 °C				
Ga partial pressure	1.39 × 10^−7^ torr				
Se partial pressure	7.76 × 10^−7^ torr				
VI/III ratio	5.6				

## Supplementary information

Supplementary information
