# Supplementary material for: Screw-Dislocation-Driven Growth Mode in Two Dimensional GaSe on GaAs(001) Substrates Grown by Molecular Beam Epitaxy
Source: Sci Rep. 2019 Nov 28;9:17781. doi: 10.1038/s41598-019-54406-5 (PMC6883029; doi:10.1038/s41598-019-54406-5)
Supplement: Supplementary file 1 — Supplementary information [file 41598_2019_54406_MOESM1_ESM.pdf]

SUPPLEMENTARY INFORMATION for

Screw-Dislocation-Driven Growth Mode in Two Dimensional GaSe on GaAs (001) Substrates grown by Molecular Beam Epitaxy

Nhu Quynh Diep[1], Cheng-Wei Liu[1], Ssu-Kuan Wu[1], Wu-Ching Chou[1,*], Sa Hoang Huynh[2,4], and Edward Yi Chang[2,3]

[1]*Department of Electrophysics, College of Sciences, National Chiao Tung University, 1001 University Road, Hsinchu 30010, Taiwan, R.O.C.*

[2]*Department of Materials Science and Engineering, College of Engineering, National Chiao Tung University, 1001 University Road, Hsinchu 30010, Taiwan, R.O.C.*

[3]*International College of Semiconductor Technology, National Chiao Tung University, 1001 University Road, Hsinchu 30010, Taiwan, R.O.C.*

[4]*Present address: School of Physics and Astronomy, Cardiff University, Cardiff CF24 3AA, United Kingdom.*

[*]***Corresponding author:*** *wuchingchou@mail.nctu.edu.tw*

Figure S1. Sketch of the growth process of without (a) and with (b) Se-pretreated samples

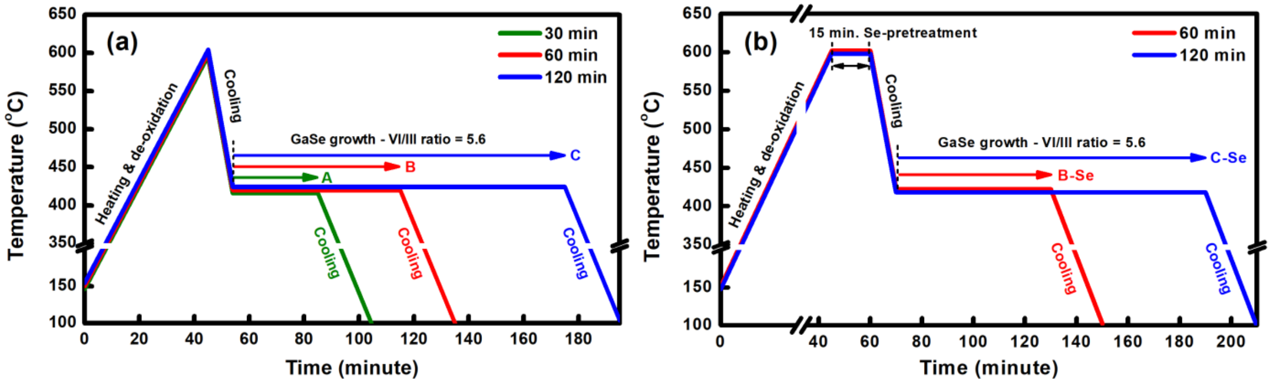

Figure S2. In-situ RHEED patterns: (a) and (b) represented to GaAs(001) substrate after thermal oxide desorption; (c) and (d) represented to non Se-pretreated sample during the materials growth; (e) the intensity profiles extracted from (c) and (d); and (f) the sketch of atomic alignment between GaSe layer and GaAs(001).

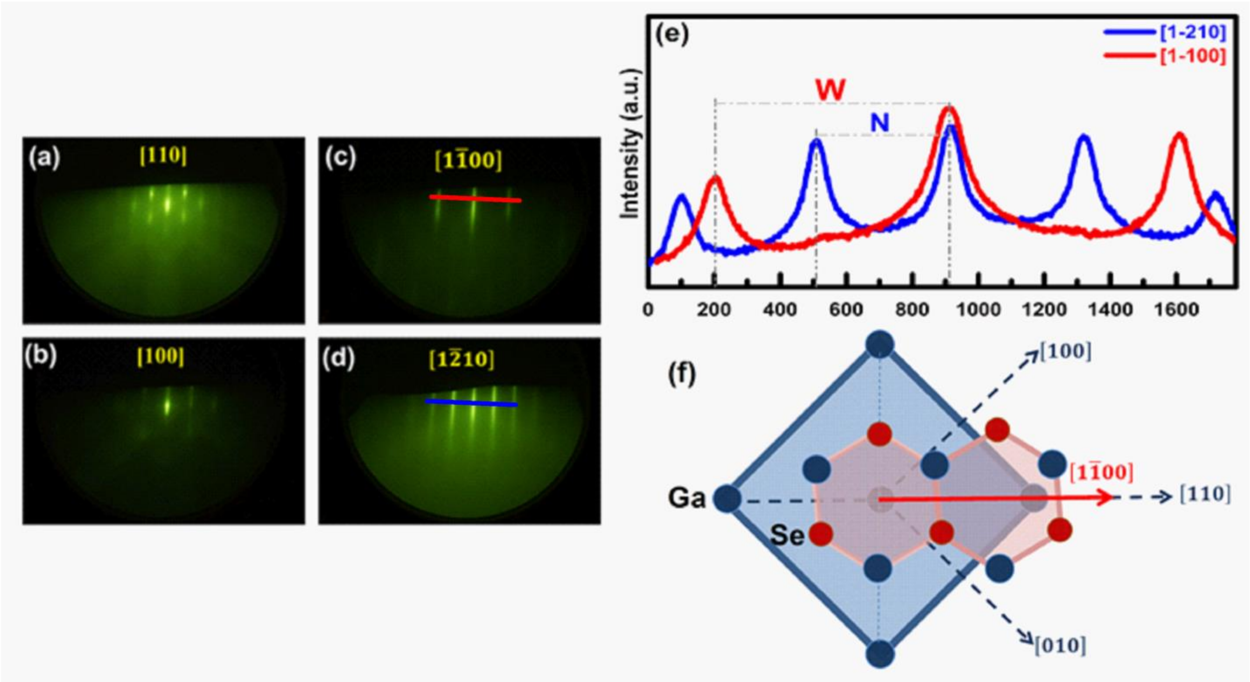

Figure S3. AFM images, Raman scattering, and photoluminescence spectrum of GaSe grown on GaN/Sapphire substrate

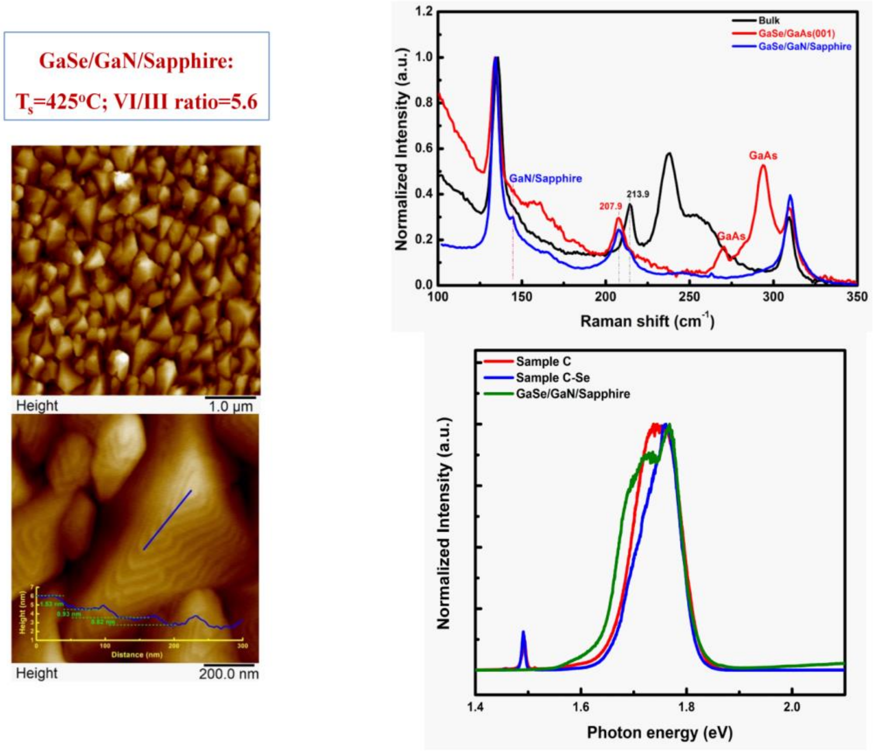

Figure S4. XRD 2θ-scanning spectra of GaSe sample C in comparison to GaSe bulk. The inset shows the 2θ-scanning spectra of (004)-peak with the step scan size of 14 arcsec.

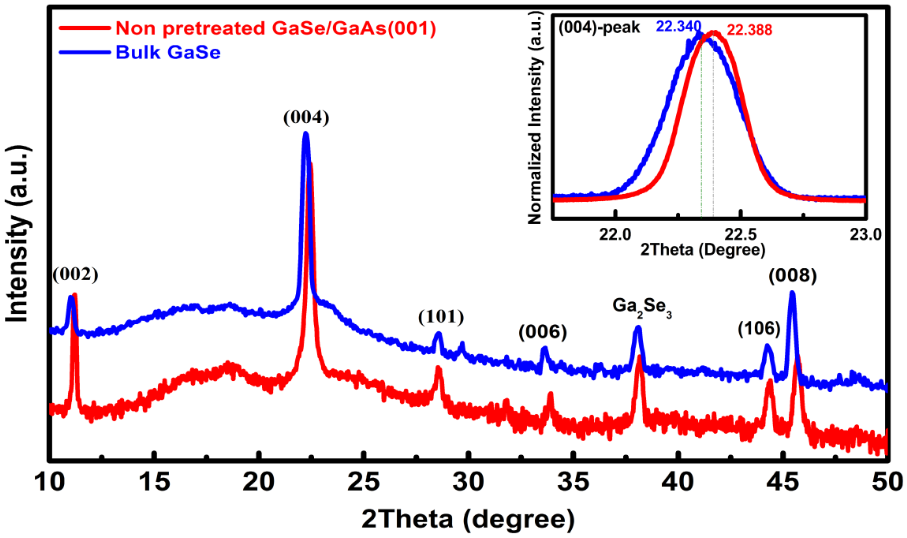

Figure S5. Raman spectrum of sample C compare to that of the bulk under 514 nm wavelength excitation.

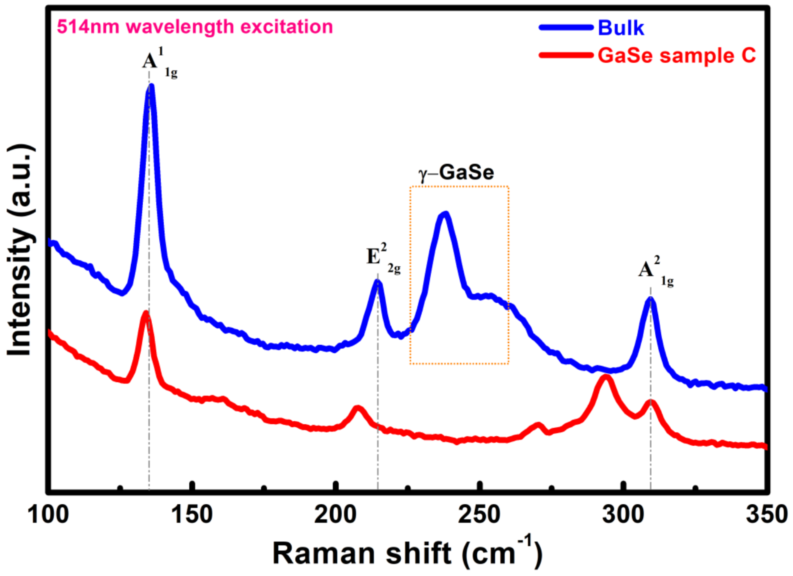

Figure S6. In-situ RHEED patterns: (a) and (b) represented to Se-pretreated GaAs(001) substrate; (c), (d) represented to Se-pretreated sample C-Se during the material growth; (e) the sketch of atomic alignment between GaSe layer and the Se-passivated GaAs(001) substrate.

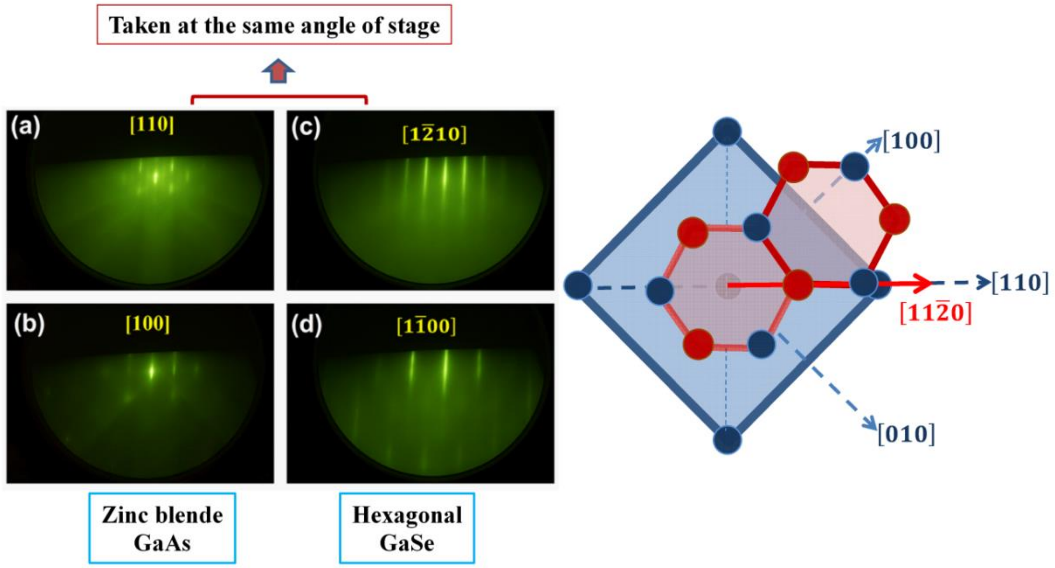

In comparison to the non Se-pretreated GaAs surface (Fig. S2a), the RHEED pattern of the Se-pretreated GaAs surface (Fig. S6a) shows extra lines in between spotty lines of GaAs. It is attributed to the presence of Se on GaAs surface.
